# Basic fibroblast growth factor sustained-release system promotes neurogenesis and tissue repair after spinal cord injury

**DOI:** 10.4103/NRR.NRR-D-25-00371

**Published:** 2025-11-25

**Authors:** Xuyang Fu, Hongmei Duan, Boya Zhang, Huan Wang, Yulin Bi, Miaoxin Yu, Peng Hao, Jian Sun, Dapeng Li, Yudan Gao, Wen Zhao, Xiaoxuan Liu, Zhaoyang Yang, Xiaoguang Li

**Affiliations:** 1 Department of Neurobiology, School of Basic Medical Sciences, Capital Medical University, Beijing, China; 2 Beijing Key Laboratory of Mental Disorders National Clinical Research Center for Mental Disorders & National Center for Mental Disorders, Beijing Anding Hospital, Capital Medical University, Beijing, China; 3 Advanced Innovation Center for Human Brain Protection, Capital Medical University, Beijing, China; 4 Department of Neurology, Peking University Third Hospital, Beijing, China; 5 Beijing Municipal Key Laboratory of Biomarker and Translational Research in Neurodegenerative Diseases, Beijing, China; 6 Department of Biomedical Engineering, School of Biological Science and Medical Engineering, Beihang University, Beijing, China

**Keywords:** basic fibroblast growth factor, chitosan, electrophysiology, endogenous regeneration, microenvironment modulation, neural stem cell, neurogenesis, single-cell RNA sequencing, spinal cord injury, sustained-release system, tissue repair

## Abstract

Spinal cord injury is accompanied by a substantial loss of neurons. Cell replacement therapy improves motor and sensory dysfunction by replacing dead neurons, and endogenous neurogenesis is an important cell replacement source. Stimulating endogenous neurogenesis is therefore a viable approach for treating spinal cord injury. Given that basic fibroblast growth factor is a potent inducer of neurogenesis, we developed a sustained-release system of basic fibroblast growth factor-chitosan to enhance tissue repair in spinal cord injury. In the present study, we isolated neural stem cells from the spinal cords of neonatal rats and used single-cell RNA sequencing to trace the complete process of neurogenesis under *ex vivo* culture conditions. Under the influence of basic fibroblast growth factor-chitosan, neural stem cells were able to transition from a quiescent state to an activated state and subsequently differentiate into neuronal precursor cells and immature neurons. Additionally, basic fibroblast growth factor-chitosan significantly enhanced neural stem cell proliferation *in vitro* and promoted neuronal generation. Subsequent *in vivo* experiments confirmed the therapeutic efficacy of basic fibroblast growth factor-chitosan in spinal cord injury, demonstrating enhanced neurogenesis and tissue repair.

## Graphical abstract

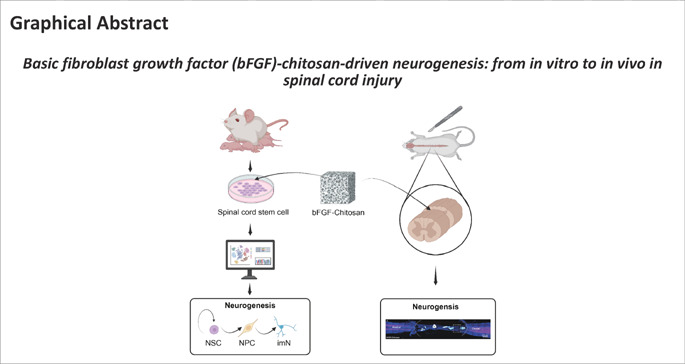

## Introduction

Spinal cord injury (SCI) is a severe neurological disorder that leads to various pathological changes, including vascular disruption, tissue swelling, acute inflammatory responses, and scar formation (Hutson and Di Giovanni, 2019). The regeneration of axons and neurons in the spinal cord is inhibited by a range of pathological changes including inflammation, edema, glial scarring, and oxidative stress; together, these result in long-lasting functional impairments in adult mammals with SCI (Silver et al., 2014). Previous studies have attempted to repair SCI through interventions such as physical therapy (Wu et al., 2020), neurotrophic factor therapy (Gao et al., 2022), and stem cell therapy (Assinck et al., 2017); however, these methods fail to promote endogenous neurogenesis and are unable to support long-distance axonal regeneration. Neurogenesis reportedly occurs after SCI, and newly generated neurons may compensate for the loss of damaged neurons (Duan et al., 2015; Yang et al., 2015; Tai et al., 2021). Additionally, newly generated neurons allow for the formation of synaptic connections with the rostral and caudal axons of the injury site, thereby restoring signal transmission (Wang et al., 2023). Promoting neurogenesis may therefore be an effective strategy for treating SCI.

Resident neural stem cells (NSCs) exist in both the spinal cord and brain. These cells possess the characteristics of self-renewal and multipotent differentiation, making them a reliable cell source for the nervous system after injury (Leiter et al., 2023; Liu et al., 2023; Zeng et al., 2023). In the early stages following SCI, a large population of activated, unidentified NSCs is present within the injured region (Xue et al., 2022). Such cellular behavior may represent a reparative response of neural tissue to injury; however, factors such as nutritional deficiencies and microenvironmental conditions can contribute to the failure of this response (Miller and Kaplan, 2012). Consequently, the majority of early-stage NSCs differentiate into astrocytes, and only a few or no NSCs differentiate into neurons (Becker et al., 2018).

Basic fibroblast growth factor (bFGF) is a member of the fibroblast growth factor family, and numerous studies have demonstrated its excellent therapeutic effects in SCI (Fan et al., 2022; Li et al., 2022; Wu et al., 2023). In neurogenesis-related studies, bFGF has been demonstrated to promote NSC activation (Carlén et al., 2009; Luo et al., 2015). Nonetheless, the poor stability, short half-life, and inactivation of bFGF within the body severely limit its application (Nguyen et al., 2013; Yu et al., 2017). Furthermore, the timing of NSC activation in response to SCI is uncertain, and a single bFGF administration does not adequately stimulate neurogenesis in the spinal cord. The development of a sustained-release system for bFGF that is capable of continuously releasing bFGF while preserving its activity might achieve favorable therapeutic outcomes in SCI.

We previously developed a sustained-release system capable of loading bFGF onto chitosan (Li et al., 2019). This sustained-release system exhibits thermal stability and provides long-term, sustained release of bFGF, thus prolonging its bioactivity. We demonstrated that bFGF-chitosan promotes the differentiation of bone marrow-derived mesenchymal stem cells and induces a high proportion of neuronal differentiation (Li et al., 2019). However, its effect on spinal cord-derived NSCs remains unknown. In the present study, we aimed to investigate whether bFGF-chitosan enhances NSC activation, neuronal differentiation, and functional maturation, and explored its potential therapeutic effects in SCI repair.

## Methods

### Construction of basic fibroblast growth factor-chitosan

Deacetylated chitosan particles (10 mg; Sigma-Aldrich, St. Louis, MO, USA) were immersed in 10 mL of deionized water for 6 hours to allow swelling. After centrifugation, the supernatant was discarded, and the hydrated particles were frozen at –20°C for 24 hours before being thawed and maintained at 4°C for 10 hours. Concurrently, bFGF (20 ng; Yisheng Biotechnology, Shanghai, China) was dissolved in 1 mL of ice-cold deionized water and gently mixed with the chitosan particles at 4°C for 6 hours. The mixture was then lyophilized under vacuum. The resulting powder was blended into a collagen I solution (Collagen Solutions, San Diego, CA, USA) with continuous stirring at 4°C for 30 minutes. Finally, the bFGF-chitosan complex was pelleted by centrifugation and stored at 4°C. For *in vitro* experiments, chitosan tubes were prepared by injecting 10 mg of the bFGF-chitosan complex into the middle of a 5-mm chitosan tube, and were maintained at 4°C.

### Culture and differentiation of spinal cord–derived neural stem cells

All experimental procedures were approved by the Experimental Animal Center of Capital Medical University and conducted in accordance with their standards (approval No. AEEI-2018-056; in 2018). Neonatal Wistar rats (24 hours old), purchased from Vital River Laboratory Animal Technology Co., Ltd. (Beijing, China; animal license No. SCXK (Jing) 2021-0006), were used for the experiments. After hypothermia-induced anesthesia, the whole spinal cord was obtained. Microforceps were used to remove the meninges and blood vessels, followed by washing with phosphate-buffered saline. Fresh cells were adjusted to a cell density of 1 × 10^5^ cells/cm^2^ in NSC proliferation medium, which consisted of Dulbecco’s modified Eagle medium/F12 (Gibco, Grand Island, NY, USA), B27 (Invitrogen, Waltham, MA, USA), bFGF (Yisheng, Beijing, China), epidermal growth factor (Tianmubeidou, Hangzhou, China), and penicillin-streptomycin (Gibco). Next, the cells were transferred to a CO_2_ incubator at 37°C for suspension culture. The cell suspension was half replaced with fresh medium every 3 days. When the NSCs had been cultured for approximately 1 week, the cells were digested with 0.25% trypsin-ethylenediaminetetraacetic acid, mechanically dissociated, and reseeded as single cells or smaller neurospheres at 1 × 10^5^ cells/cm^2^ in NSC proliferation medium for passaging. Third passage cells were obtained. Neurosphere morphology was observed using an inverted phase-contrast microscope (Leica Biosystems, Nussloch, Germany).

To induce differentiation, third passage NSCs were seeded at a density of 350 neurospheres/cm^2^ on sterile coverslips coated with poly-L-lysine. Cells in the control group were cultured with NSC differentiation medium (Dulbecco’s modified Eagle medium/F12, B27, and penicillin-streptomycin). Cells in the chitosan group were cultured in NSC differentiation medium with 10 mg/mL chitosan (Sigma-Aldrich), and cells in the bFGF-chitosan group were cultured in NSC differentiation medium with 10 mg/mL bFGF-chitosan. Half of the differentiation medium was replaced every 3 days, and differentiation markers (nestin and microtubule-associated protein 2 [MAP2]) were examined by immunofluorescence staining at 1, 3, 7, and 14 days.

### Kinetics of basic fibroblast growth factor release from basic fibroblast growth factor-chitosan

Extracellular fluid was collected from the NSCs in the bFGF-chitosan group at 1, 3, 6, and 12 hours, and weekly from 1–9 weeks. An enzyme-linked immunosorbent assay kit (Sigma-Aldrich) was used to measure bFGF release in the supernatant. The bFGF concentration was determined according to the manufacturer’s instructions. Absorbance was measured at 450 nm using a microplate reader (BMG LABTECH, Ortenberg, Germany), and the bFGF concentration was calculated based on the standard curve.

### Surgical procedure

Male Wistar rats (60 days old, weighing 250–300 g), purchased from Vital River Laboratory Animal Technology Co., Ltd. were housed in a specific pathogen-free facility under controlled environmental conditions (12-hour light/dark cycle, temperature 22 ± 2°C, relative humidity 50%–60%) with free access to standard chow and water. All rats underwent thoracic spinal cord resection at the T8 segment. The animals were randomly divided into three groups: the bFGF-chitosan group (*n* = 3) with a 5-mm-long bFGF-chitosan implant inserted into the lesion area; the lesion control group (*n* = 3) without any treatment after surgery; and the sham group (*n* = 3) with laminectomy without SCI and no further treatment. For the behavioral testing experiments, rats were again divided into three groups (*n* = 8 per group) that received the aforementioned treatments. After surgery, the bladder of each animal was manually massaged three to four times per day, and ampicillin (50 mg/day, up to 1 week; Sigma-Aldrich) was injected to prevent infection.

### 5-Bromo-2′-deoxyuridine labeling

5-Bromo-2′-deoxyuridine (BrdU; 50 mg/kg; Sigma-Aldrich) was administered via intraperitoneal injection every 12 hours for the first week after surgery. The BrdU was used to label dividing cells. The rats were sacrificed at 30 days after surgery to examine the BrdU-labeled cells.

### Immunofluorescence staining

Thirty days after surgery, three rats were randomly selected from each group and euthanized by anesthesia overdose. The animals were deeply anesthetized using an intraperitoneal injection of sodium pentobarbital (150 mg/kg; Sigma-Aldrich) until a loss of reflexes and respiration was confirmed; this was followed by transcardial perfusion. Frozen whole spinal cord tissue specimens were sectioned longitudinally or transversely using a cryostat (Leica Biosystems) to produce 8-μm sections. The sections were washed three times with 0.01 M phosphate-buffered saline, incubated overnight at 4°C with primary antibodies, and then incubated for 3 hours at room temperature (27°C) in the dark with Alexa Fluor 488/594 Affinipure goat anti-mouse IgG (1:300, Jackson Laboratory, West Grove, PA, USA, Cat# 115-545-003/115-585-003, RRID: AB_2338840/AB_2338871) and Alexa Fluor 488/594 Affinipure goat anti-rabbit IgG (1:300, Jackson Laboratory, Cat# 111-545-003/111-585-003, RRID: AB_2338046/AB_2338059). The sections were then covered with mounting medium (VectaShield, Burlingame, CA, USA) containing 4′,6-diamidino-2-phenylindole, coverslipped, and examined under a fluorescence microscope (BX-51; Olympus, Tokyo, Japan). As a control, normal goat serum (Sigma-Aldrich) was used to replace specific primary antibodies, and the same processing was performed as with the test samples. Primary antibodies included rabbit anti-nestin (1:200, Abcam, Cambridgeshire, UK, Cat# ab313787, RRID: AB_873537) or -vimentin (1:500, Abcam, Cat# ab92547, RRID: AB_10562134) for NSCs, mouse monoclonal anti-neuron-specific class III β-tubulin (Tuj1; 1:500, Abcam, Cat# ab78078, RRID: AB_2241190) for immature neurons (imNs), polyclonal rabbit anti-glial fibrillary acidic protein (GFAP; 1:1000, Abcam, Cat# ab68428, RRID: AB_11142625) for astrocytes, polyclonal rabbit anti-ionized calcium-binding adapter molecule 1 (1:500, Abcam, Cat# ab178680, RRID: AB_2832244) for microglia, polyclonal mouse anti-Ki67 (1:50, Abcam, Cat# ab15580, RRID: AB_443209) for proliferating cells, and polyclonal mouse anti-NeuN (1:500, Abcam, Cat# ab279296, RRID: AB_2895547) or rabbit anti-MAP2 (1:1000, Abcam, Cat# ab32454, RRID: AB_776174) for mature neurons. The *in vitro* experiments followed the same fixation and staining protocols. At different time points, a quantitative analysis of positive cells from the neurospheres was conducted among the three groups.

### Quantitative polymerase chain reaction

Total RNA was extracted from rat spinal cord tissue using TRIzol reagent (Invitrogen). For the lesion control and bFGF-chitosan groups, RNA was isolated from the lesion core and adjacent 5-mm segments; for the sham group, a 1-cm segment encompassing the T8–T9 region was collected. Quantitative polymerase chain reaction was performed using the primers listed in **Table 1**. Gene expression levels were analyzed using the 2^–ΔΔCT^ method (Livak and Schmittgen, 2001). All primers were designed based on gene sequences published in GenBank (https://www.ncbi.nlm.nih.gov/genbank/) using Primer Premier 5.0 software (Premier Biosoft, Palo Alto, CA, USA), and were synthesized by Sangon Biotech (Shanghai, China).

**Table 1:** Primer sequences of target genes

Gene	Forward (5'–3')	Reverse (5'–3')
*GAPDH*	AGT GCC AGC CTC GTC TCA TA	GGT AAC CAG GCG TCC GAT AC
*TNFα*	ACT GAA CTT CGG GGT GAT CG	TGG TGG TTT GCT ACG ACG TG
*IL-1β*	GGG ATG ATG ACG ACC TGC TA	ACA GCA CGA GGC ATT TTT GT
*IL-6*	TTT CTC TCC GCA AGA GAC TTCC	TGT GGG TGG TAT CCT CTG TGA

GAPDH: Glyceraldehyde-3-phosphate dehydrogenase; IL: interleukin; TNFα: tumor necrosis factor alpha.

### Single-cell RNA sequencing

The NSCs from the three groups *in vitro* were isolated into individual cells and depleted of fragmented cells, adherent cells, and dead cells under identical conditions. The cells were then processed using the Single Cell 3′ Reagent Kit v3.1 chemistry (10× Genomics, Pleasanton, CA, USA). The constructed system was sequenced using the Illumina HiSeq 2500 platform (Illumina, San Diego, CA, USA) with the Sequencing by Synthesis kit (Illumina). Unique Molecular Identifier codes were obtained using Cell Ranger (v.2.0.1) software (10× Genomics). Cell clustering and differential gene expression analysis were performed using the Seurat (v.4.3.0; Satija Lab, New York, NY, USA) package in R (v.4.3.0). The FindIntegrationAnchors and IntegrateData functions were used to integrate the data from the three groups and remove batch effects. Cell selection criteria during data processing included feature genes with expression levels > 500 and < 10,000, total gene expression levels < 50,000, and mitochondrial genes < 3% in the cells. Principal component analysis was performed using the RunPCA function in the Seurat package, and dimensionality reduction was performed using the Uniform Manifold Approximation and Projection (UMAP) method (Hao et al., 2022). The ElbowPlot function was used to determine the optimal resolution, which was set at 0.5. The FindMarkers function in the Seurat package was used to identify differentially expressed genes among cell populations using the Wilcoxon test. The DotPlot and FeaturePlot functions in the Seurat package were used to create bubble plots and gene expression plots, respectively.

The CellCycleScoring function in the Seurat package was used to score the cell cycle for each cell based on gene expression, distinguishing cells in the G1, S, and G2/M phases. The FindMarkers function in the Seurat package was used to identify stage-specific genes during NSC differentiation. The enrichGO function in the clusterProfiler software package (version 4.10.0; https://www.bioconductor.org/packages/release/bioc/html/clusterProfiler.html; Yu et al., 2012) was used for Gene Ontology (GO) enrichment analysis.

The Monocle package (v.2.28.0; Cole Trapnell Lab, University of Washington, Seattle, WA, USA) was used for the pseudo-temporal analysis of the clustered NSC, imN, and neural progenitor cell (NPC) populations identified by Seurat. The FindAllMarkers function in the Seurat package was used to identify specifically expressed genes in each cell population, and genes with an adjusted *P*-value < 0.05 were designated as defining genes for the process. The reduceDimension function was used for dimensionality reduction using the DDRTree method. The orderCells function was used to sort cells based on gene expression and create a cell distribution plot along the pseudo-temporal trajectory. The plot_genes_in_pseudotime function was used to visualize the expression changes of individual genes along the pseudo-temporal trajectory. The differentialGeneTest function was used to identify genes that changed with cell differentiation based on pseudotime values. The plot_pseudotime_heatmap function was used to create a heatmap showing gene expression patterns along the pseudo-temporal trajectory, clustered based on gene expression trends. The GO enrichment analysis and Kyoto Encyclopedia of Genes and Genomes pathway enrichment analysis were performed using the clusterProfiler software package.

### Patch clamp recording

Under an inverted microscope (DMi8, Leica Biosystems), neurons derived from *in vitro*-differentiated neurospheres were selected. A glass microelectrode (World Precision Instruments, Sarasota, FL, USA) with an impedance of 5.5–7 MΩ was used to make a tight seal with the target cell by suction. The holding potential was set at –70 mV, and capacitance compensation was applied. To isolate voltage-gated calcium currents, tetrodotoxin (1 μM; Sigma-Aldrich) was included in the extracellular solution to block sodium channels. A brief negative pressure was applied to the membrane to achieve whole-cell recording mode. Tetrodotoxin was used to block sodium channels. Data were acquired using an EPC-10 amplifier (HEKA, Lambrecht, Germany) and PATCHMASTER software (v.2.90; HEKA), and were analyzed using Clampfit (Molecular Devices, Sunnyvale, CA, USA). The Mini Analysis Program 6.0 (Synaptosoft, Fort Lee, NJ, USA) and Clampfit software (v11; Molecular Devices) were used to analyze the data.

### Behavioral assessment

Basso, Beattie, and Bresnahan Locomotor Rating Scale (BBB) scores were assessed in each group (*n* = 8 per group) at 2 days before surgery, 1 day postoperatively, and 1, 2, 4, 6, 8, 10, 12, and 24 weeks postoperatively. Hindlimb functional recovery was evaluated by observers blinded to the treatment conditions, as previously described (Basso et al., 1995; Li et al., 2009). BBB scores range from 0 (complete paralysis) to 21 (normal locomotion).

### Statistical analysis

All data are presented as the mean ± standard error of the mean (SEM), and data organization and chart generation were performed using GraphPad Prism (version 10.0.0 for Windows, GraphPad Software, Boston, MA, USA; www.graphpad.com). For data that conformed to a normal distribution, the mean values from individual groups were compared using independent, unpaired, two-tailed Student’s *t*-test and one- or two-way analysis of variance followed by Sidak’s or Bonferroni *post hoc* tests for multiple comparisons. For data that did not conform to a normal distribution, we used Steel–Dwass *post hoc* tests or Kruskal–Wallis tests for comparisons. The *P*-value was set as < 0.05.

## Results

### Basic fibroblast growth factor-chitosan promotes neural stem cell differentiation into neurons *in vitro*

**Figure 1:**
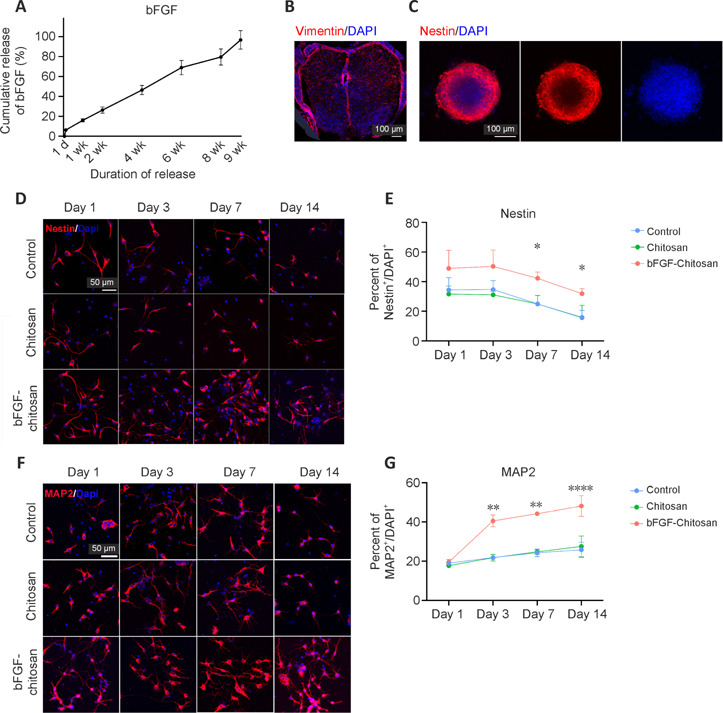
bFGF-chitosan promotes NSC differentiation into neurons *in vitro*. (A) bFGF was continuously released *in vitro* from chitosan carriers loaded with bFGF (20 ng/mL) for at least 9 weeks (*n* = 6 neurospheres). (B) Confocal immunofluorescence image of neonatal rat NSCs. Coronal sections of the spinal cord were stained for the NSC marker vimentin (red; Alexa Fluor 594) and DAPI (blue). Scale bar: 100 μm (*n* = 3). (C) Confocal immunofluorescence image of neurospheres, which were stained for nestin (red; Alexa Fluor 594) and DAPI (blue). Scale bar: 100 μm. (D–G) Confocal immunofluorescence images of neurospheres during *in vitro* culture, which were stained with nestin (red; Alexa Fluor 594) or MAP2 (red; Alexa Fluor 594) (nestin: Day 1: *n* = 5/control, *n* = 3/chitosan, *n* = 4/bFGF-chitosan; Day 3: *n* = 6/control, *n* = 3/chitosan, *n* = 3/bFGF-chitosan; Day 7: *n* = 3/control, *n* = 3/chitosan, *n* = 4/bFGF-chitosan; Day 14: *n* = 4/control, *n* = 4/chitosan, *n* = 4/bFGF-chitosan; MAP2: Day 1: *n* = 4/Control, *n* = 4/Chitosan, *n* = 3/bFGF-chitosan; Day 3: *n* = 3/control, *n* = 3/chitosan, *n* = 3/bFGF-chitosan; Day 7: *n* = 3/control, *n* = 3/chitosan, *n* = 3/bFGF-chitosan; Day 14: *n* = 5/control, *n* = 4/chitosan, *n* = 5/bFGF-chitosan). Over time, the bFGF-treated group exhibited decreased numbers of stem cells (nestin^+^) alongside increased neuronal differentiation (MAP2^+^). Scale bars: 50 μm. Data are presented as the mean ± SEM. *P < 0.05, **P < 0.01, ****P < 0.0001, *vs*. control group (one- or two-way analysis of variance followed by Sidak’s or Bonferroni *post hoc* tests). bFGF: Basic fibroblast growth factor; Dapi: 4’,6-diamidino-2-phenylindole; MAP2: microtubule-associated protein 2; NSCs: neural stem cells.

Chitosan, a natural polysaccharide derived from chitin, has been widely used as a drug delivery carrier owing to its excellent biocompatibility, biodegradability, low toxicity, and controlled-release properties. Based on these characteristics, chitosan was selected as the carrier material for constructing the bFGF sustained-release system in this study. We first cultured NSCs with bFGF-chitosan and measured the extracellular concentration of bFGF at different time points to determine its release profile. bFGF-chitosan sustained the release of bFGF for up to 9 weeks (**Figure 1A**), demonstrating its ability to achieve stable, prolonged release.

Fan et al. (2022) have identified specific cells in the spinal cord that may exhibit NSC characteristics. However, the exact origin of these cells remains unclear. Vimentin (a marker of NSCs (Fan et al., 2022)) was used to identify NSCs at 24 hours post-birth; NSCs in the rats were predominantly located in the spinal cord ependyma (**Figure 1B**). To assess whether bFGF-chitosan can promote the differentiation of NSCs into neurons, NSCs were isolated and cultured from the spinal cords of 24-hour-old rats. The identity of cells within the neurospheres was confirmed using the NSC marker nestin (**Figure 1C**). After 1, 3, 7, and 14 days of induction, immunofluorescence staining was performed to quantify the number of nestin^+^ NSCs and MAP2^+^ neurons (MAP2 specifically labels neuronal cell bodies and dendrites (Sánchez et al., 2000)). With induction culture, the number of NSCs continuously decreased (**Figure 1D** and **E**), indicating ongoing differentiation, whereas the number of mature neurons slowly increased over time (**Figure 1F** and **G**). Compared with the other groups, the bFGF-chitosan group exhibited more MAP2^+^ neurons, indicating that bFGF-chitosan promotes the neuronal differentiation of NSCs. Additionally, there was no significant difference in the number of NSCs induced by the empty carrier compared with the control group, suggesting that chitosan has no cytotoxicity to NSCs.

### Single-cell RNA sequencing of spinal cord–derived neurospheres

**Figure 2:**
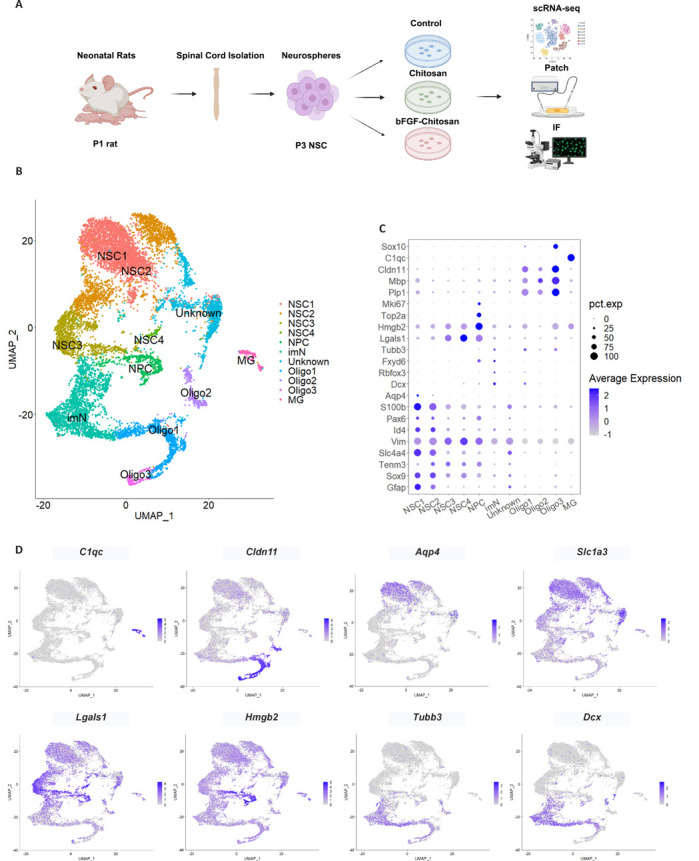
scRNA-seq analysis reveals the characteristics of specific molecules in neurospheres. (A) Experimental flowchart. Created with BioRender.com. (B) UMAP visualization of 11 cell clusters comprising 11,742 cells from neurospheres and their progeny derived from three cultures. (C) Expression of the discriminative marker genes in the 11 cell populations. (D) UMAP visualizations, colored using the expression of known marker genes for the selected cell population. imN: Immature neuron; MG: microglia; NPC: neural progenitor cell; NSC: neural stem cell; Oligo: oligodendrocyte; scRNA-seq: single-cell RNA sequencing; UMAP: Uniform Manifold Approximation and Projection.

We investigated the effects of bFGF-chitosan on NSCs during neurogenesis. Specifically, we explored the stages at which bFGF-chitosan promotes NSC differentiation, and the types of cells that are generated (**Figure 2A**). 14 days after the initiation of differentiation at passage 3, single-cell RNA sequencing (scRNA-seq) results revealed a dataset comprising 3082 cells in the control group, 4825 cells in the chitosan group, and 3835 cells in the bFGF-chitosan group. Unsupervised clustering and UMAP visualization of the dataset revealed 11 clusters (**Figure 2B**). These clusters were identified using classical markers, and encompassed all NSCs and their derivative cell populations as well as a small portion of other cell types (**Figure 2C** and **D**). Specifically, the identified cell clusters included: (1) neuroglia, consisting of Oligo1 (*Sox10*^low+^ *Plp1*^+^ *Mbp*^+^*Cldn11*^+^), Oligo2 (*Plp1*^+^ *Mbp*^+^*Cldn11*^+^), Oligo3 (*Sox10*^high+^ *Plp1*^+^ *Mbp*^+^*Cldn11*^+^), and a limited number of infiltrating microglia (*C1qc*^+^). (2) NSC1 (partially overlapping with *Aqp4*^+^ astrocytes) and NSC2 (*Slc1a3*^+^ and *Lgals1*^−^); NSC3 and NSC4 (*Slc1a3*^+^ and *Lgals1*^+^); NPCs (*Hmgb2*^+^); imN (*Dcx*^+^*Tubb3*^+^); and other unknown cells.

### Identification of the differentiation process from neural stem cells to immature neurons

**Figure 3:**
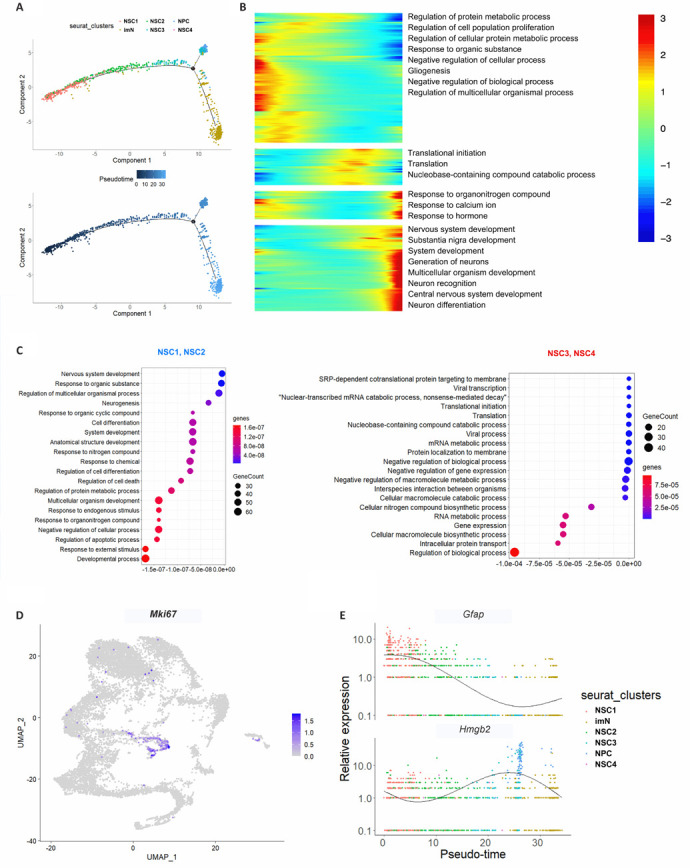
Comparative analysis of neurogenesis-related clusters identifies key transcriptional changes during neural differentiation. (A) Pseudotime trajectory of cells in the neurospheres. (B) DEGs along the trajectory. Left: Heatmap of the average expression of each gene along the trajectory and neurogenesis stages. Right: Enriched biological pathways. (C) GO terms of DEGs between NSC1/NSC2 and NSC3/NSC4. Selected GO and KEGG terms that were significantly enriched for NSC1/NSC2 and NSC3/NSC4 are shown. (D) UMAP visualizations, colored by the expression of *Mki67* for the selected cell population. (E) *Gfap* and *Hmgb2* gene expression distributions of NSCs, NPCs, and imNs. DEGs: Differentially expressed genes; GFAP: glial fibrillary acidic protein; GO: Gene Ontology; Hmgb2: high-mobility group protein B2; imNs: immature neurons; KEGG: Kyoto Encyclopedia of Genes and Genomes; Mki67: marker of proliferation Ki-67; NPCs: neural progenitor cells; NSCs: neural stem cells; UMAP: Uniform Manifold Approximation and Projection.

To determine the cellular identities during the differentiation of NSCs into imNs, pseudotime analysis was performed to calculate the trajectories of NSC1, NSC2, NSC3, NSC4, NPC, and imN, with an aim to infer the differentiation trajectory between these clusters. The origin trajectory of NSCs initiated from NSC1 and progressed through NSC2, NSC3, NSC4, and NPC, ultimately leading to imNs (**Figure 3A**). This finding supports our identification of the differentiation process from NSCs to imNs.

We next analyzed differentially expressed genes along the pseudotime trajectory, and observed distinct enrichment patterns in each stage (**Figure 3B**). In the early NSC stage, genes were mainly enriched in cellular proliferation and related biological processes. In the NPC stage, genes were primarily enriched in protein translation and associated processes. In the imN stage, genes were predominantly enriched in neurodevelopment and neuronal generation processes.

NSC1 comprises two populations: astrocytes and NSCs. Given the shared expression of multiple similar gene types between these two populations, they were unable to be separated. NSC1 and NSC2 express several NSC marker genes, such as *Gfap*, *Slc4a4*, *Slc1a3*, and *Pax6* (Hao et al., 2022; **Figure 2C**). Additionally, NSC1 and NSC2 exhibit quiescent NSC-related marker genes, including *S100*β, *Id4*, and *Sox9* (Shah et al., 2018; Shu et al., 2022). NSC3 and NSC4 express the NSC marker gene *Slc1a3* as well as the activated NSC marker gene *Hmgb2*, which is a chromatin-associated transcriptional activator that is critical for transitioning NSCs from quiescent to proliferative (Kimura et al., 2018). Additionally, they express Lgals1, which is a gene that is specifically upregulated in spinal cord stem/progenitor cells after SCI (Hao et al., 2022; Shu et al., 2022).

To determine the identities of NSC1, NSC2, NSC3, and NSC4, we compared the differentially expressed genes between NSC1/NSC2 and NSC3/NSC4 (**Figure 3C**). NSC1 and NSC2 exhibited high expression of *Fxyd1*, *Hspb1*, *Clu*, and *Apoe*. Functional analysis indicated that the specifically expressed genes were mainly enriched in processes such as neurodevelopmental responses to organic substances, regulation of multicellular organism processes, neurogenesis, responses to organic cyclic compounds, development of cell differentiation systems, anatomical structure development in response to nitrogen compounds, responses to chemicals, and regulation of cell differentiation. These findings suggest that NSC1 and NSC2 possess functional characteristics that align more closely with quiescent NSCs. By contrast, NSC3 and NSC4 showed high expression of *Igfbp2*, *Ppp1r14b*, *Hnrnpa1*, *Polr2f*, *Gap43*, *Lmo1*, *Tpm1*, *Rps12*, and *Rps9*. Functional analysis revealed that these genes were mainly enriched in processes related to mRNA metabolism, macromolecular metabolic processes, protein transport, and protein translation. Previous studies have indicated that ribosomal biogenesis genes are selectively upregulated during the transition from quiescent to activated NSCs (Shin et al., 2015; Chen et al., 2017). The enrichment analysis of NSC3 and NSC4 supported the enrichment of processes related to ribosomes, thereby supporting the identification of NSC3 and NSC4 as activated NSCs.

NPCs represent a highly proliferative cell population that expresses many genes related to the cell cycle, such as *Mki67* and *Top2a* (**Figure 3D**). Additionally, it has been reported that in the subgranular zone, *GFAP* expression gradually decreases during the differentiation of radial glia-like cells into NPCs, whereas *HMGB2* expression increases (Hao et al., 2022). In NSC development, we observed a similar phenomenon (**Figure 3E**). During the transition from NSC1 to NPC, *Gfap* expression gradually decreased, whereas *Hmgb2* expression increased.

### Basic fibroblast growth factor-chitosan promotes neurogenesis by activating neural stem cells

**Figure 4:**
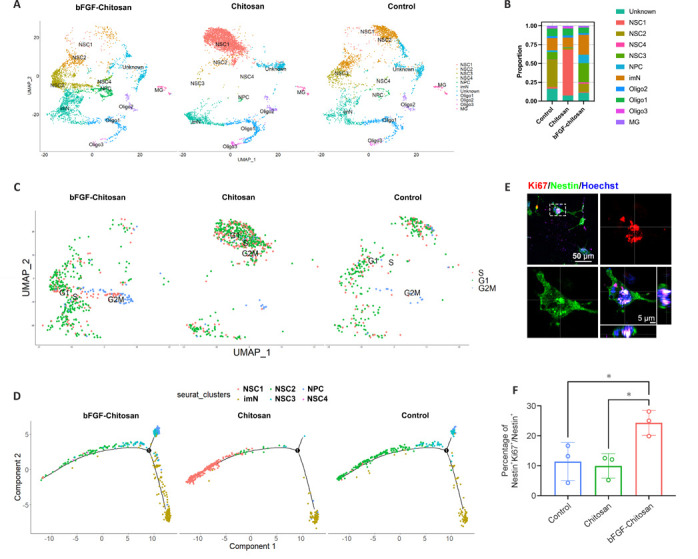
bFGF-chitosan persistently activates NSCs. (A) UMAP plot of the control, chitosan, and bFGF-chitosan datasets. (B) Proportion of each type of cell. (C) Cell cycle status (G1 and G2/M phases). (D) Pseudotime trajectory of cells. (E) Confocal immunofluorescence images of cells in the bFGF-chitosan group at 14 days of *in vitro* culture; the cells were stained with nestin (green; Alexa Fluor 488) and Ki67 (green; Alexa Fluor 488). Scale bars: 50 μm, 5 μm (enlarged images). (F) Quantitative analysis of nestin^+^Ki67^+^ cells at 14 days. Data are presented as the mean ± SEM (*n* = 3 independent neurospheres). *P < 0.05 (one-way analysis of variance). bFGF: Basic fibroblast growth factor; imN: immature neuron; MG: microglia; NPC: neural progenitor cell; NSC: neural stem cell; Oligo: oligodendrocyte; UMAP: Uniform Manifold Approximation and Projection.

We analyzed the diversity of cell types in the control, chitosan, and bFGF-chitosan groups (**Figure 4A**). All cell types were present in all groups (**Figure 4B**), indicating that the bFGF-chitosan-induced activation of NSCs does not lead to exhaustion. However, there were significant changes in the numbers of specific cell types. Although there were similar numbers of oligodendrocytes in all three groups, we observed more NSC1 and NSC2 in the control and chitosan groups, and more NSC3, NPCs, and imNs in the bFGF-chitosan group. This suggests that in the control and chitosan groups, NSCs may predominantly remain in the quiescent NSC state, with a higher rate of differentiation into astrocytes and oligodendrocytes. Conversely, in the bFGF-chitosan group, more NSCs enter the activated NSC state and show a higher propensity for neuronal generation.

We then performed cell cycle analysis on cells from different groups. NSCs in the bFGF-chitosan group exhibited higher expression of G2/M genes in cells near the tip of the NPC population (**Figure 4C**). Most NPCs expressed G2/M phase genes, whereas few cells expressed G2/M genes in the other groups. This suggests that bFGF-chitosan has a sustained release effect, allowing NSCs to continuously enter an activated state. Even after 14 days of culture in the conditional medium, a relatively large number of NSCs were still in the proliferative stage.

Through a pseudotime analysis (**Figure 4D**), we identified similar developmental trajectories among the different groups. Additionally, we observed that only a limited number of NSCs in the control and chitosan groups entered the neuronal development program, whereas bFGF-chitosan promoted the differentiation of NSC1 and NSC2 into activated NSC stages. This sustained activation led to the continuous development of more cells into imNs.

We then conducted a 14-day *in vitro* cell experiment under different treatment conditions. After 14 days of neurosphere culture, only a few cells in the control and chitosan groups were Ki67^+^nestin^+^ (proliferating cell markers). By contrast, the bFGF-chitosan group exhibited more Ki67^+^nestin^+^ cells (**Figure 4E** and **F**), indicating that bFGF-chitosan significantly promotes NSC proliferation.

**Figure 5:**
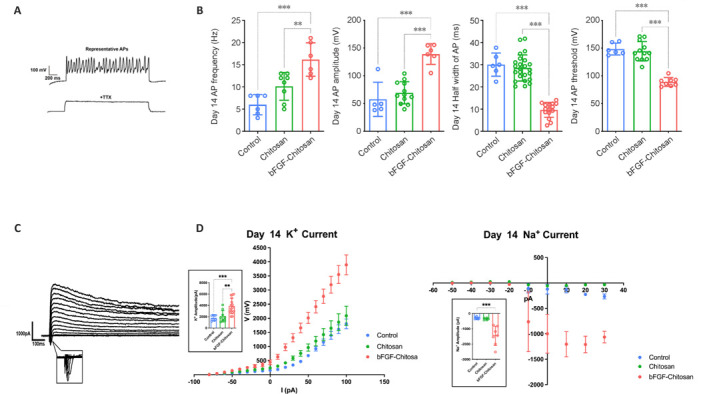
Neurons display a more mature phenotype in culture with bFGF-chitosan. (A) Representative trace of AP firing without or with TTX (to block AP) triggered by current injection at 14 days *in vitro*. (B) AP frequency (*n* = 6/control, *n* = 8/chitosan, *n* = 6/bFGF-chitosan), AP amplitude (*n* = 5/control, *n* = 12/chitosan, *n* = 6/bFGF-chitosan), width at half-maximal amplitude (*n* = 5/control, *n* = 12/chitosan, *n* = 6/bFGF-chitosan), and AP threshold (*n* = 6/control, *n* = 12/chitosan, *n* = 8/bFGF-chitosan) were calculated. (C) Representative voltage-clamp recordings showing inward Na^+^ currents followed by outward K^+^ currents at 14 days *in vitro*. (D) Voltage-current relationships for K^+^ (*n* = 8/control, *n* = 9/chitosan, *n* = 15/bFGF-chitosan) and Na^+^ (*n* = 7/control, *n* = 6/chitosan, *n* = 7/bFGF-chitosan) currents at 14 days *in vitro*. Data are presented as the mean ± SEM. **P < 0.01, ***P < 0.001 (one- or two-way analysis of variance followed by Sidak’s or Bonferroni *post hoc* tests). AP: Action potential; bFGF: basic fibroblast growth factor; TTX: tetrodotoxin.

To evaluate the functional maturity of neurons generated through continuous neurogenesis under bFGF-chitosan stimulation, we performed membrane patch clamp electrophysiology on neurons at 14 days *in vitro* under different treatment conditions to measure their action potentials and characterize their excitability. Neurons induced by bFGF-chitosan exhibited higher action potential frequencies and amplitudes compared with the other two control groups (**Figure 5A** and **B**). They also had shorter half-widths and lower threshold currents (**Figure 5A** and **B**), indicating a higher level of functional maturity in the bFGF-chitosan group. To assess the contribution of sodium (Na^+^) and potassium (K^+^) channels during the maturation process, we used voltage clamp techniques to examine neurons. Neurons induced by bFGF-chitosan generated larger inward (I)Na and IK currents (**Figure 5C** and **D**). Collectively, these results suggest that neurons induced by bFGF-chitosan exhibit a relatively high level of maturity.

### Basic fibroblast growth factor-chitosan improves the spinal cord microenvironment after spinal cord injury

**Figure 6:**
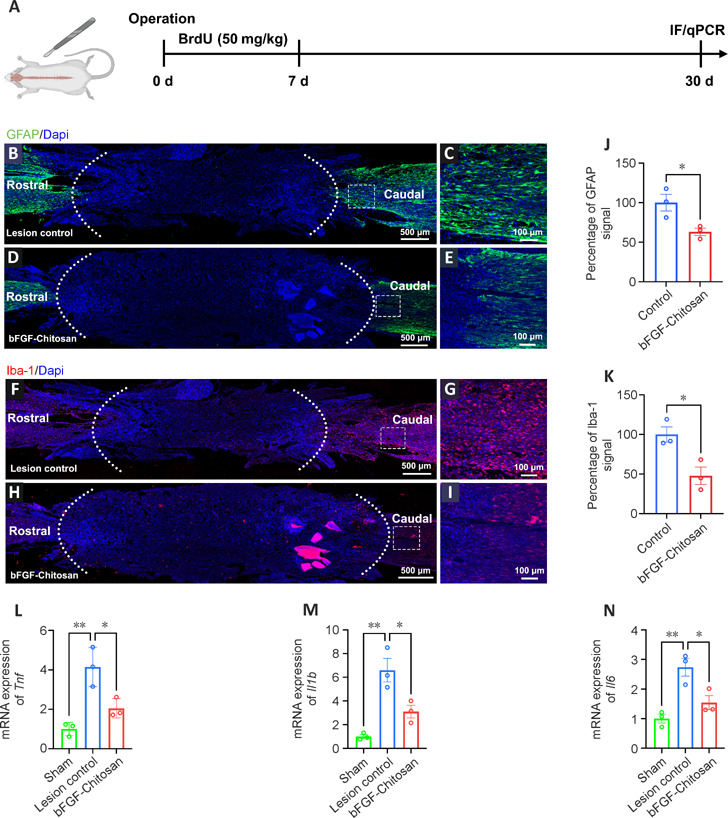
bFGF-chitosan improves the microenvironment in the lesion area after SCI. (A) Experimental flowchart, created with BioRender.com. (B–I) Confocal immunofluorescence images of GFAP^+^ (green; Alexa Fluor 488) and Iba-1^+^ (red; Alexa Fluor 594) cells at 30 days after SCI (*n* = 3). bFGF treatment attenuated the infiltration of both reactive astrocytes and activated microglia compared with the lesion control group. (J, K) Fluorescence intensity of GFAP and Iba-1 in spinal cord tissue at 30 days after SCI (*n* = 3). (L–N) Quantitative analysis of *Tnf*, *Il1b*, and *Il6* mRNA expression in spinal cord tissue at 30 days after SCI (*n* = 3). Data are expressed as the mean ± SEM. *P < 0.05, **P < 0.01 (one-way analysis of variance). bFGF: Basic fibroblast growth factor; Dapi: 4′,6-diamidino-2-phenylindole; GFAP: glial fibrillary acidic protein; Iba-1: ionized calcium-binding adapter molecule 1; IF: Immunofluorescence; IL: interleukin; qPCR: quantitative polymerase chain reaction; SCI: spinal cord injury; TNFα: tumor necrosis factor alpha.

Previous studies have reported limited neurogenesis in the adult spinal cord (Habib et al., 2016; Tai et al., 2021; Shu et al., 2022), and the post-injury microenvironment remains a major obstacle to neural repair. Notably, chitosan exerts potent anti-inflammatory effects and suppresses glial scar formation (Duan et al., 2015; Yang et al., 2015; Zhao et al., 2022). To evaluate the internal environment provided by bFGF-chitosan after SCI, we used a T8–T9 complete spinal cord transection model (**Figure 6A**). In the lesion control group at 30 days post-SCI, astrocytes were activated, resulting in the formation of glial scars on both sides of the lesion area. Simultaneously, a chronic inflammatory response characterized by microglial activation and proliferation and astrocytic proliferation was observed (**Figure 6B**, **C**, **F**, and **G**). However, the immediate implantation of bFGF-chitosan after SCI significantly inhibited scar formation (**Figure 6D**, **E**, and **J**) and attenuated microglial activation (**Figure 6H**, **I**, and **K**).

To further investigate the changes in spinal cord inflammation, we used a T8 complete spinal cord transection model. At 30 days post-injury, we dissected the lesion core and adjacent 5-mm segments of spinal cord tissue from the injured groups, and a 1-cm segment encompassing the T8–T9 region from the sham group. Quantitative polymerase chain reaction was performed to assess the expression of pro-inflammatory cytokines, including Tnf, Il1b, and Il6. Compared with the sham group, the lesion control group exhibited markedly elevated Tnf, Il1b, and Il6 expression (**Figure 6I–N**), indicating a persistent chronic inflammatory response at 30 days post-injury. Notably, the bFGF-chitosan group exhibited significantly reduced expression of these inflammatory mediators relative to the lesion control group (**Figure 6I–N**). These findings further indicate that bFGF-chitosan implantation effectively attenuates chronic inflammation in the spinal cord, thereby contributing to the establishment of a favorable microenvironment for neurogenesis following SCI.

### Basic fibroblast growth factor-chitosan improves functional recovery after spinal cord injury through neurogenesis

**Figure 7:**
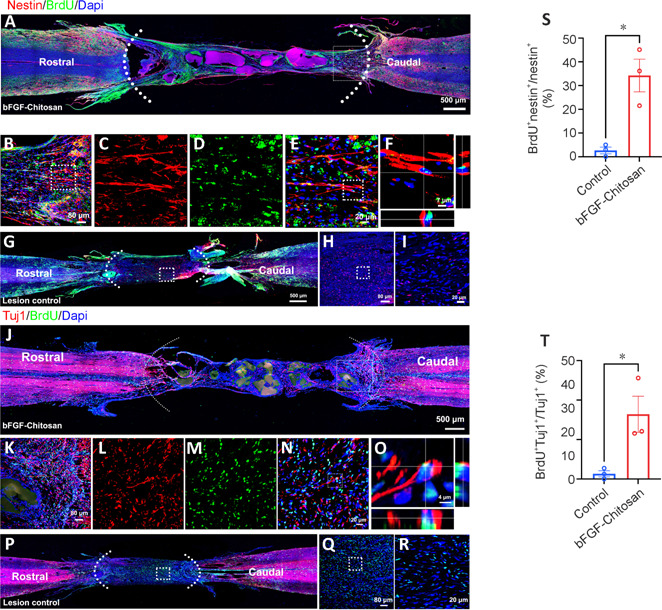
bFGF-chitosan promotes endogenous neurogenesis in the lesion area after SCI. (A–F) Confocal immunofluorescence images of BrdU-labeled (green; Alexa Fluor 488) and nestin^+^ (red; Alexa Fluor 594) cells in nerve tissue from the bFGF-chitosan group at 1 month after SCI (*n* = 3). Some nestin^+^ cells exhibited BrdU incorporation (green; Alexa Fluor 488) in their nuclei. (G–I) Confocal immunofluorescence images of BrdU-labeled and nestin^+^ cells in the injured/regenerating nerve tissue of the control group at 1 month after SCI (*n* = 3). (J–O) Confocal immunofluorescence images of BrdU-labeled and Tuj1^+^ (red; Alexa Fluor 594) cells in the injured/regenerating nerve tissue of the bFGF-chitosan group at 1 month after SCI (*n* = 3). (P–R) Confocal immunofluorescence images of BrdU-labeled and Tuj1^+^ cells in the nerve tissue of the control group at 1 month after SCI (*n* = 3). (S, T) Quantitative analysis of BrdU^+^nestin^+^/nestin^+^ and BrdU^+^Tuj1^+^/Tuj1^+^ (*n* = 3). All data are expressed as the mean ± SEM. *P < 0.05 (independent, unpaired, two-tailed Student’s *t*-test). bFGF: Basic fibroblast growth factor; BrdU: 5-bromo-2’-deoxyuridine; Dapi: 4′,6-diamidino-2-phenylindole; SCI: spinal cord injury; Tuj1: neuron-specific class III β-tubulin.

**Figure 8:**
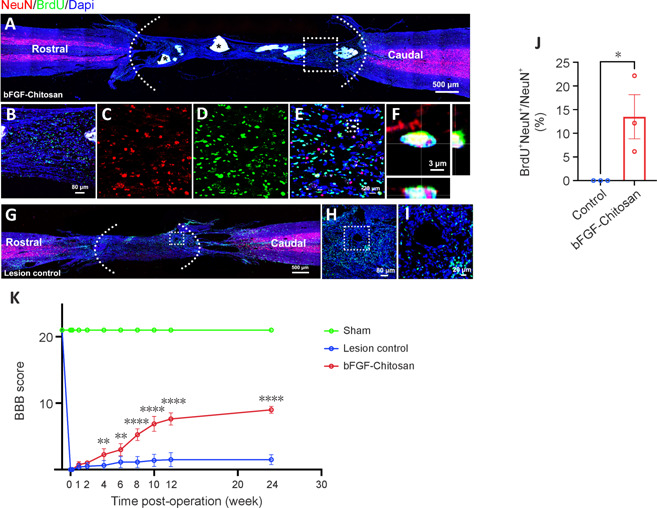
bFGF-chitosan promotes adult neural stem cells to differentiate into mature neurons after SCI. (A–F) Confocal immunofluorescence images of BrdU-labeled and NeuN^+^ cells in the injured/regenerating nerve tissue of the bFGF-chitosan group at 1 month after SCI (*n* = 3). (G–I) Confocal immunofluorescence images of BrdU-labeled and NeuN^+^ cells in the injured/regenerating nerve tissues of the control groups at 1 month after SCI (*n* = 3). (J) Quantitative analysis of BrdU^+^NeuN^+^/NeuN^+^ (*n* = 3). (K) BBB scores (*n* = 8). All data are expressed as the mean ± SEM. *P < 0.05, **P < 0.01, ****P < 0.0001, *vs*. lesion control group (two-way analysis of variance followed by Sidak’s or Bonferroni *post hoc* tests). BBB: Basso-Beattie-Bresnahan; BrdU: 5-bromo-2′-deoxyuridine; bFGF: basic fibroblast growth factor; Dapi: 4′,6-diamidino-2-phenylindole; NeuN: neuronal nuclei; SCI: spinal cord injury.

In our initial investigation, we explored NSC proliferation. We identified virtually no actively dividing NSCs (nestin^+^/BrdU^+^) within the injured area 30 days post-injury in the lesion control group (**Figure 7G–I**). By contrast, the introduction of bFGF-chitosan led to a substantial surge in NSC proliferation (BrdU^+^/nestin^+^) within the injured region (**Figure 7A–F** and **S**), offering strong evidence for the pronounced enhancement of NSC proliferation by bFGF-chitosan. We also observed that 30 days post-injury, there were no imNs (BrdU^+^/Tuj1^+^) present in the lesion control group (**Figure 7P–R**). However, bFGF-chitosan implantation facilitated the differentiation of NSCs into imNs (BrdU^+^/Tuj1^+^) (**Figure 7J–O** and **T**). A small portion of these imNs further matured into functional neurons (BrdU^+^/NeuN^+^) in the bFGF-chitosan group (**Figure 8A–F** and **J**), whereas no neurons were observed in the lesion control group. These results suggest that bFGF-chitosan can activate NSCs to differentiate into mature neurons.

To evaluate whether bFGF-chitosan facilitates functional recovery, we quantitatively assessed hindlimb motor function using the BBB scoring system. At 1-day post-injury, both the lesion control and bFGF-chitosan groups exhibited a complete loss of hindlimb motor function, with BBB scores reduced to 0. Over time, gradual increases in BBB scores were observed in both groups. By week 1 post-injury, slight movements of the hip and knee joints were occasionally observed in some rats from both groups. By week 2, rhythmic but limited joint activity emerged, which progressed to mild multi-joint movements by week 4. Notably, from weeks 4–12, motor function in the lesion control group plateaued, with no significant improvements. Long-term observation revealed that joint contractures and increased muscle tone restricted movement, and beyond 12 weeks, rats in the lesion control group exhibited only residual movement in one to two joints and displayed a characteristic dorsal paw-dragging gait, with BBB scores stabilizing at 2–3 points (**Figure 8K**). By contrast, rats in the bFGF-chitosan group showed significantly greater functional recovery. At week 8, rats in this group exhibited large-range joint movements; by week 10, they began to drag their hindlimbs during forward locomotion; and by week 24, BBB scores reached 9–10, with some rats displaying weight-supported hindlimb movement (**Figure 8K**). Together, these results indicate that bFGF-chitosan implantation can promote marked spontaneous motor function recovery following complete SCI.

## Discussion

It remains uncertain whether NSCs exist in the spinal cord, although many studies have demonstrated their presence using *in vitro* culture techniques. However, most studies have identified cell types using immunofluorescence staining, which is a method that may present issues such as nonspecific labeling and false positives. Moreover, *in vitro* cell culture can involve reprogramming or trans-differentiation rather than neurogenesis, which may lead to the expression of neuronal markers. It thus remains to be determined whether these cells possess the full functionality of neurons. New technologies are therefore needed to provide more compelling evidence for the existence of NSCs. In recent years, scRNA-seq has provided new insights, offering robust evidence for neurogenesis (Hao et al., 2022; Zhou et al., 2022). In the present study, we used scRNA-seq to confirm the presence of NSCs with neurogenic potential in the spinal cord and to trace the entire neurogenesis process. We conducted a detailed investigation of cells committed to a neuronal fate and demonstrated that the differentiation process from NSCs to imNs exhibits similarities to that of other classical neurogenic regions (Zhou et al., 2022). For example, NSC activation has been observed to involve the upregulation of ribosome biogenesis, which promotes NSC activation (Hetman and Slomnicki, 2019; Farooq et al., 2020).

In the current study, we used classical markers to identify astrocytes, oligodendrocytes, and imNs, and demonstrated the differentiation potential of ependymal cells from the spinal cord of neonatal rats into astrocytes, oligodendrocytes, and neurons. Furthermore, we observed a cluster of oligodendrocytes, and previous studies have demonstrated the potential of ependymal cells to differentiate into oligodendrocytes, even in adult rats (Llorens-Bobadilla et al., 2020), thus providing a clue to the origin of NSCs from the ependyma.

Previous studies have demonstrated that bFGF promotes neurogenesis (Xiao et al., 2007; Ravin et al., 2008; Zhou et al., 2018). In the present study, we used bFGF-chitosan as a sustained-release carrier to enhance neurogenesis. scRNA-seq analysis revealed that bFGF-chitosan activates more NSCs into activated NSCs, which then proliferate as NPCs (Shin et al., 2015), indicating that bFGF primarily drives neurogenesis by NSC activation. Furthermore, the quantity of oligodendrocytes remained similar across the different groups in the current study, suggesting that bFGF-chitosan does not disrupt NSC differentiation toward the oligodendrocyte lineage. To assess the maturity of these neurons, we performed patch clamp recordings. Although there were differences in electrophysiological properties compared with mature neurons *in vivo*, the neurons in the bFGF-chitosan group displayed a more mature phenotype (Bai et al., 2023). These differences might be attributed to variations in culture time and the culture environment.

In the current study, we demonstrated that bFGF-chitosan synergistically promotes neurogenesis in SCI models through complex molecular and cellular interactions. These studies will not only provide valuable information about NSC biology but may also offer important insights for neurogenesis-based therapeutic strategies. However, several limitations must be acknowledged. First, although primary spinal cord NSCs remain among the most physiologically relevant models for studying neurogenesis post-SCI, *in vitro* culture systems cannot fully recapitulate the *in vivo* microenvironment, in which extracellular cues and cell–cell interactions play critical roles. Second, we exclusively used male rats to avoid the potential confounding effects of estrogen cycles on spinal injury pathology. However, we recognize that sex differences may affect specific recovery mechanisms, and future studies should therefore include female animals to validate the broader applicability of our findings. Third, although we observed that bFGF-chitosan enhances neurogenesis by increasing NSC proliferation, the precise molecular mechanisms remain incompletely understood. Prior studies suggest that bFGF may act through the Janus kinase/signal transducer and activator of transcription and phosphoinositide 3-kinase/protein kinase B pathways (Numakawa and Kajihara, 2023); however, whether the bFGF-chitosan combination engages additional or novel signaling pathways requires further investigation. Addressing these questions will be essential for fully elucidating the therapeutic potential of this approach.

## Data Availability

*No additional data are available.*
